# Can functioning scores guide rehabilitation referral in primary care? Extending ClinFIT to the community level

**DOI:** 10.3389/fresc.2026.1770872

**Published:** 2026-04-23

**Authors:** Fatimah Ahmedy, Mohd Nazri Mohd Daud, Sukhbeer Kaur Darsin Singh, Frisca A. Francis, Candace Goh, Julia Patrick Engkasan, Melissa Selb, Gerold Stucki

**Affiliations:** 1 Sabah Rehabilitation Research & Service Group, Faculty of Medicine & Health Sciences, Universiti Malaysia Sabah, Kota Kinabalu, Malaysia; 2 Department of Family Medicine, Faculty of Medicine & Health Sciences, Universiti Malaysia Sabah, Kota Kinabalu, Malaysia; 3 Department of Nursing, Faculty of Medicine & Health Sciences, Universiti Malaysia Sabah, Kota Kinabalu, Malaysia; 4 Department of Rehabilitation Medicine, Hospital Universiti Malaysia Sabah, Kota Kinabalu, Malaysia; 5 Physiotherapy Unit, Beaufort Hospital, Beaufort, Malaysia; 6 Department of Rehabilitation Medicine, Faculty of Medicine, Universiti Malaya, Kuala Lumpur, Malaysia; 7 ICF Research Branch, Swiss Paraplegic Research, Nottwil, Switzerland; 8 Department of Health Sciences & Health Policy, Faculty of Health Sciences & Medicine, University of Lucerne, Lucerne, Switzerland

**Keywords:** functioning assessment, functioning-based triage, primary care, rehabilitation referral, ICF

## 1 Introduction

The global need for rehabilitation is substantial and continues to grow. Recent estimates from the Global Burden of Disease study indicate that approximately 2.4 billion people, around one in three individuals worldwide, live with health conditions that could benefit from rehabilitation services at some point during the course of their illness or injury (1). Primary care is often the first point of contact for individuals seeking healthcare and typically includes services delivered by general practitioners, community-based physiotherapists, and other first-contact healthcare providers depending on the health system context. Yet referral decisions still rely largely on diagnosis, impairment severity, and informal clinical judgement rather than standardised measures of functioning (2). This mismatch contributes to under-referral, delayed referral, and inequitable access to rehabilitation, particularly in low- and middle-income countries.

The International Classification of Functioning, Disability and Health (ICF), developed by the World Health Organization (WHO), is a comprehensive framework for describing and measuring health and disability (3). Within the ICF, functioning refers to the dynamic interaction between a person's health condition and contextual factors, encompassing body functions and structures, activities, participation, and environmental influences. The ICF therefore provides a common language for describing how health conditions affect individuals' ability to function in daily life across different settings and populations. Building on this framework, the International Society of Physical and Rehabilitation Medicine developed the Clinical Functioning Information Tool (ClinFIT) as a pragmatic approach to capture functioning data in routine practice (4). ClinFIT sets derived from the ICF Generic-30 have been validated primarily in inpatient rehabilitation and specialist services, demonstrating feasibility, responsiveness, and clinical utility for documenting functioning profiles, monitor rehabilitation outcomes, and support clinical decision-making and care planning (5).

More recently, ClinFIT has been applied to specific populations and clinical questions. These include developing COVID-19–specific item sets (6), staging cut-off scores for rehabilitation provision (7) and intensity, and condition-specific versions for musculoskeletal disorders (8). These initiatives show that functioning scores can be linked to meaningful clinical decisions. However, these have largely been confined to hospitals or specialist rehabilitation settings. The potential for using functioning scores as a basis for *triage and referral* at the population level, meaning the systematic identification of rehabilitation needs among individuals presenting to primary care services, remains underexplored.

In this article, we outline the rationale and a proposed screening tool, termed the ClinFIT Primary Care Screening Questionnaire, a functioning-based triage tool designed to support early identification of rehabilitation needs in primary care. The proposed tool aims to guide referral decisions from primary care to appropriate rehabilitation services, which may include community-based physiotherapy services, hospital outpatient rehabilitation programs, or multidisciplinary rehabilitation teams depending on the organisation of the health system. By operationalising functioning assessment within routine primary-care practice, this approach aims to support more consistent and transparent rehabilitation referral pathways across health systems.

Therefore, this article aims to describe the adaptation of the ClinFIT for use in community and primary care settings, and to outline a validation pathway for its use as a screening method to support functioning-based rehabilitation referral, with the intention of stimulating future empirical research and validation studies.

## 2 Lessons from existing ClinFIT research for referral decision-making

The original ClinFIT initiative was conceived as a universal, ICF-based tool that could be applied across health conditions and along the continuum of care (4). Evidence from inpatient rehabilitation settings has demonstrated that ClinFIT scores change meaningfully over the course of rehabilitation and follow-up, with medium to large, standardised response means (5). This supports the responsiveness of the tool to clinically important changes and its usefulness in monitoring rehabilitation outcomes in routine practice.

A recent study further extended this work by deriving functional staging cut-off scores based on ClinFIT summary scores (7). By linking functioning scores with patterns of service use and rehabilitation intensity, this work proposed score ranges corresponding to different rehabilitation needs, such as intensive inpatient rehabilitation, less intensive outpatient programs, or primarily self-management support. These thresholds illustrate how functioning scores may inform clinical decision-making related to rehabilitation planning.

In addition, condition-specific adaptations of ClinFIT, such as the version developed for musculoskeletal disorders (8), demonstrate the flexibility of the tool. These adaptations show that selected subsets of ClinFIT items can capture key domains of functioning relevant to specific clinical contexts while maintaining alignment with the broader ICF framework.

Taken together, these findings indicate that ClinFIT scores are psychometrically robust, responsive to clinical change, and capable of supporting clinical decision-making. They also highlight the potential for strategic item selection to tailor the tool for different healthcare contexts. Building on this evidence, the present article explores the possibility of adapting selected ClinFIT items to support triage and rehabilitation referral decisions within primary care settings.

## 3 Why functioning-based thresholds are needed in primary care

Primary care systems vary considerably across countries, but they commonly serve as the first point of contact for individuals seeking healthcare. In many developing and middle-income countries, including Malaysia, referral decisions regarding rehabilitation services are often initiated by primary care clinicians, such as general practitioners or family medicine specialists, who assess patients during routine consultations. In contrast, in many high-income countries, rehabilitation decisions may involve multidisciplinary teams, including physiotherapists, occupational therapists, and rehabilitation physicians working collaboratively.

Nevertheless, across health systems, identifying rehabilitation needs early remains challenging because referral decisions frequently rely on clinical judgment and diagnosis rather than standardised measures of functioning. Given that rehabilitation interventions must be tailored to individuals' specific functional limitations and participation restrictions, structured approaches to identifying rehabilitation needs in primary care may improve referral consistency and equity.

Several ICF-based instruments already exist that could inform primary-care decision-making. The ICF Generic-30 (Rehabilitation) Set and the ICF Generic-7 (Minimal Generic Set) provide concise sets of categories that capture key domains of functioning across health conditions (9, 10). Studies in China, Japan, Nepal and other settings have demonstrated the feasibility of using minimal ICF sets to screen for disability, document functioning profiles, and support planning in routine services (11–13).

However, these initiatives generally stop short of defining explicit score thresholds linked to referral pathways. Screening results may prompt further clinical assessment, but the relationship between specific scores and decisions such as “refer to multidisciplinary rehabilitation” or “manage within primary care” is rarely formalised. As a result, functioning data are at risk of remaining descriptive rather than decisional.

In health systems where rehabilitation resources are limited and unevenly distributed, explicit functioning-based thresholds anchored in a standardised tool could make referral decisions more transparent and equitable, help prioritise patients with the greatest functioning loss or risk of long-term disability, provide a population-level overview of rehabilitation needs within primary-care catchment areas, and facilitate clearer communication between primary-care providers and rehabilitation teams through the use of a shared language of functioning.

## 4 A Proposal: the ClinFIT Primary Care Screening Questionnaire

Building on the established evidence base supporting ClinFIT's clinical utility in rehabilitation medicine, we propose the development of a ClinFIT Primary Care Screening Questionnaire, a short, functioning-based triage instrument derived exclusively from selected items within the ClinFIT-30. This adapted tool aims to support population-level identification of rehabilitation needs in primary care, where early recognition can prevent long-term disability yet often remains under-prioritised.

The ClinFIT-30 item set, derived from the ICF Generic-30 Set, is a clinical functioning assessment tool designed to capture key domains of functioning across health conditions. It measures aspects of body functions, activities, and participation that are relevant to rehabilitation and has been primarily applied in inpatient and specialist rehabilitation settings. ClinFIT-30 is typically used by rehabilitation clinicians to document functioning profiles, monitor functional change over time, and support rehabilitation planning and outcome evaluation. Two main features render ClinFIT-30 as an optimal foundation for developing a primary-care referral tool:

- **Universality**
  The items capture common functioning issues seen across chronic, acute, and multimorbid presentations, which are typical of primary-care caseloads.
- **Demonstrated clinical relevance**
  Prior studies have shown that ClinFIT scores correlate well with rehabilitation intensity, resource use, and meaningful change over time, supporting its sensitivity to clinical needs.

These characteristics facilitate the development of a streamlined tool that remains conceptually anchored in ICF/ClinFIT while being feasible for routine use in fast-paced primary-care settings.

### 4.1 Strategic item selection from ClinFIT-30

The ClinFIT Primary Care Screening Questionnaire would not use all 30 items; instead, it would identify a concise set of high-yield items that most effectively discriminate between patients who require referral and those who do not, while representing a balanced range of functioning domains such as mobility, daily activities, pain, emotional functioning, and participation. These selected items would reflect the clinical reasoning rehabilitation physicians apply when determining rehabilitation needs and would remain feasible for rapid administration by non-specialist primary-care providers.

The proposed item selection strategy is informed by established principles used in the development and validation of clinical screening instruments and ICF-based measurement tools. These approaches aim to ensure that the resulting questionnaire is both psychometrically robust and feasible for routine use in primary-care settings. Item selection would be guided by:

- ROC analysis comparing item ratings to rehabilitation physician judgement.
- Single-item predictive accuracy (sensitivity, specificity, AUC values).
- Construct-item analysis to ensure coverage of multiple domains.
- Feasibility data from pilot testing.

### 4.2 Scoring and administration

To maximise clarity and ease of use in primary care settings, the ClinFIT Primary Care Screening Questionnaire will adopt an intuitive 0–10 numeric rating scale instead of the traditional ICF 0–4 qualifiers (5, 14). Numeric rating scales are widely used in clinical practice, particularly in patient-reported outcome measures, where patients are asked to rate symptoms or functional difficulty on a simple numerical continuum. This format has been extensively applied in areas such as pain assessment, fatigue measurement, and functional limitation, and has demonstrated good validity, reliability, and responsiveness across a range of clinical populations (15–17).

Compared with categorical scales, numerical rating scales offer several practical advantages. They are easy to understand for patients with different educational and health literacy backgrounds, require minimal explanation, and can be administered quickly during routine consultations (15, 16). The 0-10 format also allows finer gradations of perceived difficulty, which may improve sensitivity in capturing subtle differences in functioning or monitoring changes over time. In addition, such scales are already familiar to both patients and clinicians because they are commonly used in routine clinical assessment, thereby reducing cognitive burden and facilitating integration into busy primary-care workflows (16, 17).

Scoring involves deriving a total score by summing the ratings from the selected items depending on the final subset chosen. Higher total scores indicating greater functioning difficulty and, consequently, a higher likelihood of rehabilitation need. This scoring approach preserves the conceptual fidelity of the ClinFIT framework while enhancing the practicality and psychosocial acceptability of the questionnaire for population-level screening in primary care settings.

### 4.3 Deriving functioning-based referral thresholds

To transform functioning scores into actionable triage decisions, a phased psychometric evaluation is proposed:

#### 4.3.1 Gold-standard classification: rehabilitation physicians independently classify patients into

- *Requires referral to rehabilitation services*
- *Does not currently require rehabilitation referral*

#### 4.3.2 Threshold development

ROC-derived cut-off scores will identify the total score ranges that best distinguish these categories. Optimal thresholds will balance sensitivity and specificity for primary-care use.

#### 4.3.3 Item-level optimisation

- Predictive validity of each item
- Impact of removing/adding specific items
- Internal consistency of the reduced item set

### 4.4 Implementation pathway

The proposed ClinFIT Primary Care Screening Questionnaire represents a reduced-item instrument derived from selected items within the ClinFIT-30, rather than a direct adaptation of the full ClinFIT-30 tool. The aim is to develop a concise screening questionnaire that retains the conceptual foundation of ClinFIT while focusing on a subset of high yield functioning domains that are most relevant for identifying rehabilitation needs in primary care.

This partciular questionnaire can be operationalised through a four-phase strategy:

#### 4.4.1 Adaptation

Identifying and refining a reduced subset of ClinFIT-30 items suitable for primary care use, including translating and adapting item wording to ensure cultural and linguistic appropriateness. Cognitive interviews with clinicians will be conducted to ensure that the selected items are clear, relevant, and interpreted as intended.

#### 4.4.2 Pilot testing

Assessing the feasibility of administering the questionnaire in real clinical settings, including evaluating administration time and overall user acceptability among patients and providers. Based on these findings, the layout, formatting, and scoring instructions would be refined to ensure clarity, efficiency, and ease of use in primary-care workflows.

#### 4.4.3 Large-scale validation study

Deriving functioning-based cut-off scores through ROC analysis to determine optimal thresholds for rehabilitation referral, while also confirming the construct validity of the reduced item set to ensure it accurately reflects the core dimensions of functioning relevant to rehabilitation needs in primary care.

#### 4.4.4 Clinical integration evaluation

Embedding the tool into routine primary-care workflows to ensure it is used consistently during patient assessments, while simultaneously monitoring its impact on referral patterns, equity of access to rehabilitation services, and overall service utilisation.

## 5 Discussion

While functioning assessment is well established within rehabilitation medicine, its systematic use to support referral triage in primary care remains limited. Most existing ClinFIT applications focus on outcome monitoring within specialist rehabilitation settings. The present proposal therefore extends the framework upstream in the care pathway, aiming to support earlier identification of rehabilitation needs at the point of first healthcare contact.

Using functioning scores to guide rehabilitation referral in primary care is, in our view, a logical extension of the ICF and ClinFIT agendas. It aligns with global calls to strengthen rehabilitation within health systems rather than limiting rehabilitation services to specialised settings, as highlighted in international initiatives such as the World Health Organization's Rehabilitation 2030 agenda (1, 18).

Several challenges must be acknowledged. Previous studies examining the integration of rehabilitation services within health systems have highlighted barriers such as limited rehabilitation workforce capacity, variability in referral practices across primary care settings, and concerns among clinicians regarding additional documentation burden in routine clinical workflows (19–21). Addressing these challenges will require careful implementation strategies, including clinician engagement, integration with existing clinical systems, and ongoing evaluation of feasibility and impact.

Despite these caveats, the potential benefits are considerable. A functioning-based referral tool could help identify hidden rehabilitation needs, support advocacy for resources by quantifying unmet demand, and provide a common language linking primary care, rehabilitation services and health policy. It would also position ClinFIT not only as an outcome measure within rehabilitation, but as a strategic instrument for *rehabilitation planning at the population level*.

We contend that functioning scores derived from subsets of ClinFIT items can and should be used to guide rehabilitation referral at the population level in primary care. The time is right to move from conceptual alignment between ICF and primary care to practical tools that support real-world decisions, and the ClinFIT Primary Care Screening Questionnaire represents one promising step in that direction.

The proposed ClinFIT Primary Care Screening Questionnaire offers one pathway to operationalise this vision. By focusing on a minimal set of core functioning domains and using simple rating scales that may incorporate patient-reported perceptions of functioning difficulty, the proposed questionnaire aims to balance conceptual rigor with real-world feasibility in primary-care settings. Importantly, it does not replace clinical judgement; rather, it provides a structured input into decision-making, supporting more consistent and transparent referrals.

Future work should therefore prioritise collaborative, multi-country projects that test variations of this screening questionnaire in diverse primary-care settings, particularly in low- and middle-income countries where rehabilitation gaps are greatest. Such projects could build directly on the growing body of ClinFIT research, leveraging existing expertise while shifting the focus upstream, from rehabilitation wards to the communities where people live and seek first-line care. We invite rehabilitation clinicians, researchers, and international partners to join in testing, refining, and scaling this approach across diverse health systems.
